# METTL3 mediates atheroprone flow–induced glycolysis in endothelial cells

**DOI:** 10.1073/pnas.2424796122

**Published:** 2025-05-06

**Authors:** Guo-Jun Zhao, So Yun Han, Yajuan Li, Dongqiang Yuan, Shuo Qin, Yuhan Li, Hongje Jang, Li-Jing Chen, Tong-You Wade Wei, Ming He, Yi-Shun Li, Zhen Bouman Chen, Lingyan Shi, Shu Chien, John Y-J Shyy

**Affiliations:** ^a^Department of Cardiology, The First Affiliated Hospital of Zhengzhou University, Zhengzhou 450052, China; ^b^Division of Cardiology, Department of Medicine, University of California, La Jolla, CA 92093; ^c^Department of Bioengineering, University of California at San Diego, La Jolla, CA 92093; ^d^Institute of Engineering in Medicine, University of California, La Jolla, CA 92093; ^e^Department of Diabetes Complications and Metabolism, Beckman Research Institute, City of Hope, Duarte, CA 91010

**Keywords:** shear stress, endothelial cell, glycolysis, atherosclerosis

## Abstract

Atheroprone flow–increased glycolysis in vascular endothelial cells (ECs) is pivotal in EC dysfunction and the initiation of atherosclerosis. Methyltransferase 3 (METTL3) is a major m^6^A methyltransferase for RNA N6-mehtyladenosine (m^6^A) modifications to regulate epitranscriptome and cellular functions. With the atheroprone flow upregulating METTL3 and m^6^A RNA hypermethylation, we investigate the role of METTL3 in atheroprone flow–induced glycolysis in ECs in vitro and in vivo. Compared to pulsatile shear stress (PS, atheroprotective flow), oscillatory shear stress (OS, atheroprone flow) increases METTL3 expression to enhance the m^6^A modifications of mRNAs encoding HK1, PFKFB3, and GCKR, which are rate-limiting enzymes of glycolysis. These augmented m^6^A modifications increase the expressions of HK1 and PFKFB3 while decreasing GCKR, resulting in elevated EC glycolysis, as revealed by seahorse analysis. Moreover, a stimulated Raman scattering (SRS) imaging study demonstrates the elevation of glucose incorporation into de novo synthesized lipids in ECs under atheroprone flow in vitro and in vivo. Empagliflozin, a sodium-glucose cotransporter-2 inhibitor (SGLT2i) drug, represses METTL3 expression, thereby mitigating OS-induced glycolysis in ECs. These data suggest mechanisms by which METTL3 links EC mechanotransduction with metabolic reprogramming under atherogenic conditions.

## Significance

The atheroprone flow–increased glycolysis contributes to endothelial dysfunction and atherosclerosis. This study demonstrated that atheroprone flow increases endothelial glycolysis via METTL3. The induction of METTL3 promotes the m^6^A methylation of key glycolysis genes, HK1, PFKFB3, and GCKR. Conversely, atheroprotective flow and SGLT2i Empagliflozin reduce EC glycolysis via suppressing METTL3 and m^6^A methylation of HK1, PFKFB3, and GCKR. These flow-regulated metabolic changes can be attenuated genetically by the manipulation of METTL3 or pharmacologically using SGLT2i. Thus, our data provide insights into mechanotransduction linked to metabolism in EC health and disease.

The atheroprone flow–induced endothelial cell (EC) dysfunction plays a pivotal role in the initiation and progression of atherosclerosis (1). In vivo and in vitro, atheroprone flow pattern induces the expression of proinflammatory and proliferative genes, along with metabolic reprogramming in ECs (2–4). In principle, glycolytic genes, e.g., hexokinases (HKs) and 6-phosphofructo-2-kinase/fructose-2,6-biphosphatase 3 (PFKFB3), are induced, while glucokinase regulatory protein (GCKR) is suppressed, under atheroprone flow (4–6). Thus, glycolysis is increased in ECs under atheroprone flow to meet the increased energy demand for the proinflammatory and proliferative phenotype (7). Such Warburg effect-like increase in glycolysis constitutes a part of EC dysfunction, leading to atherogenesis (3, 4).

Epitranscriptomes, i.e., RNA modifications without altering the nucleotide sequence, emerge as crucial mechanisms regulating gene expression (8–10). As the most abundant RNA epitranscriptomic modification in eukaryotes, N^6^-methyladenosine (m^6^A) is tightly regulated by m^6^A methyltransferases, binding proteins, and demethylases, which are referred to as m^6^A writers, readers, and erasers, respectively (11). Ample evidence shows that m^6^A RNA modifications are widely involved in EC biology and disease (12, 13). Methyltransferase 3 (METTL3), a major m^6^A methyltransferase, has been implicated in diverse cellular functions and disease processes, including glycolysis (14–16). We have previously demonstrated that METTL3 and RNA m^6^A modifications are induced by oscillatory shear stress (OS, mimicking atheroprone flow) in ECs (2). Silencing METTL3 in ECs abrogates the NLR family pyrin domain-containing 1 (NLRP1)-mediated inflammasome and monocyte adhesion induced by atheroprone flow, whereas METTL3 overexpression inhibits the atheroprotective flow–induced KLF4 (2). In vivo, METTL3 knockdown suppresses atheroprone flow–induced stenosis in partially ligated mouse carotid arteries (2). These findings suggest that METTL3 plays a critical role linking mechanotransduction with EC dysfunction. However, whether METTL3 is involved in OS-induced glycolysis in ECs remains unclear.

Cardiovascular drugs can exert EC protection through molecular mechanisms similar to those mediated by PS (1, 17). For example, sodium-glucose cotransporter 2 inhibitors (SGLT2i), an FDA-approved medication to treat diabetes, have demonstrated profound beneficial effects on EC biology and atherosclerosis, in part through the inhibition of glycolysis (18). However, whether such inhibition of EC glycolysis by SGLT2i is mediated via a METTL3-regulated epitransciptome remains to be determined.

This study tests the hypothesis that atheroprone flow upregulates METTL3 to mediate m^6^A modification of glycolytic genes in ECs, thus contributing to EC dysfunction. Our results demonstrate that OS induces METTL3, which enhances the m^6^A modification of PFKFB3, HK1, and GCKR, which are key enzymes and regulators of glycolysis. Additionally, our findings reveal that SGLT2i Empagliflozin reduces glycolysis by downregulating METTL3 in ECs. Using stimulated Raman scattering (SRS) imaging to track D7-glucose (D7-glu) uptake in human ECs and mouse aortic trees, we further show that atheroprone flow increases glycolysis in a METTL3-dependent manner.

## Results

### OS Increases Glycolysis via METTL3.

**Fig. 1.**
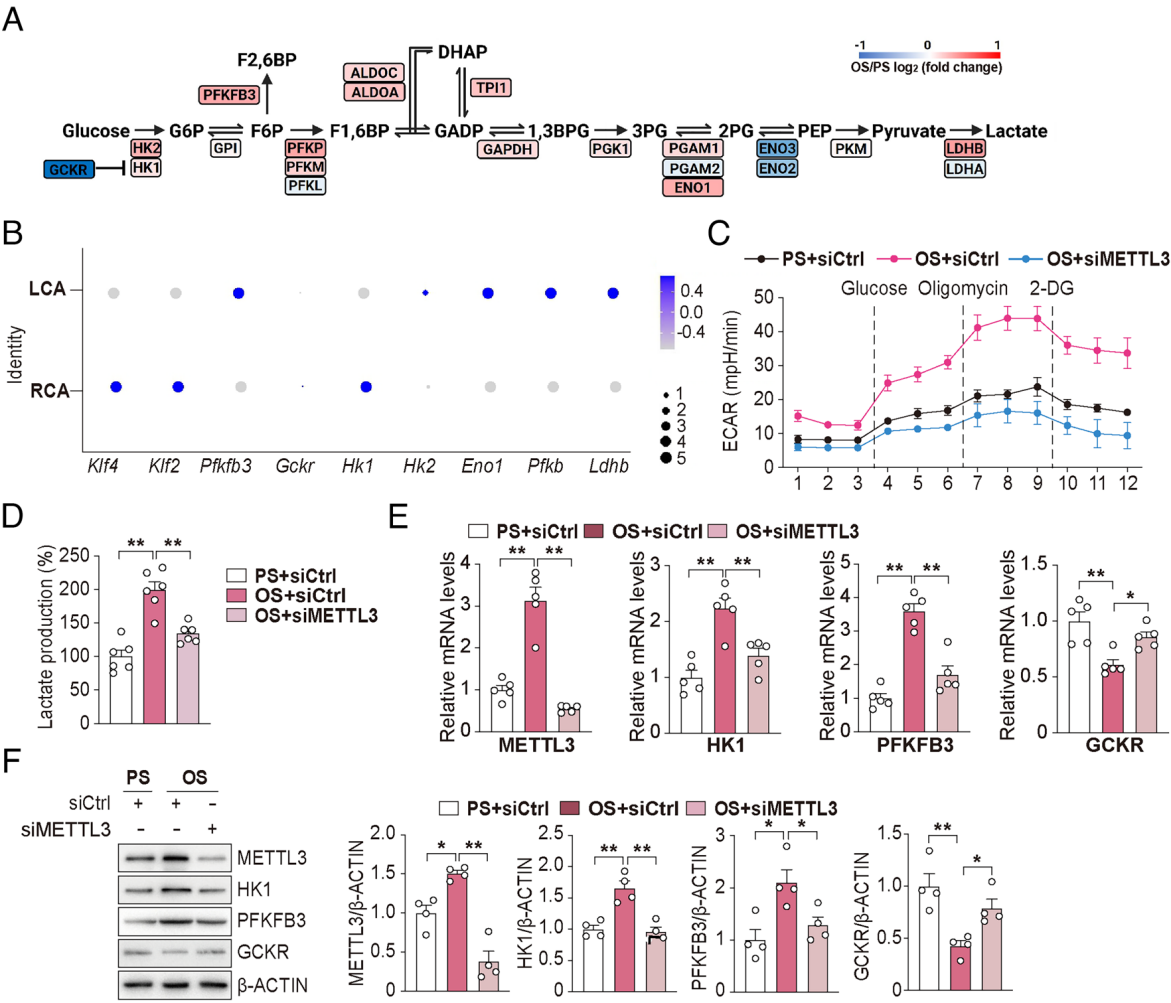
OS increases glycolysis via METTL3. (*A*) Heatmap of glycolytic gene expression based on RNA-seq analysis of ECs subjected to OS (0.5 ± 4 dyn/cm^2^) or PS (12 ± 4 dyn/cm^2^) for 48 h. The color bars represent OS/PS log2 (fold change). (*B*) scRNA-seq analysis demonstrating upregulation of glycolytic genes PFKFB3, HK2, ENO1, PFKP, and LDHB in the partially ligated left carotid artery (LCA) compared to the control right carotid artery (RCA). (*C*) Seahorse assay demonstrating the extracellular acidification rate (ECAR) in the indicated groups (n = 9/group). (*D*) Measurement of relative lactate levels in ECs under PS, OS, and OS with METTL3 knockdown (n = 6/group). (*E*) qPCR analysis of the indicated glycolytic genes (n = 5/group). (*F*) Western blot and quantification of METTL3, HK1, PFKFB3, and GCKR expressions in ECs subjected to PS or OS, with or without METTL3 knockdown (n = 4/group). Data are presented as mean ± SEM. *P < 0.05, **P < 0.01.

To identify glycolysis genes differentially regulated by OS versus PS, we first analyzed RNA-seq data (GSE103672) from ECs subjected to OS and PS (19). Compared to PS, OS upregulated most enzymes involved in glycolysis, e.g., *HK1* and *2*, *PFKFB3*, *ENO1,* and *LDHB* (Fig. 1*A*). We also analyzed a scRNA-seq dataset obtained from mouse partial carotid ligation experiment (20). Consistently, the glycolytic genes, including *Hk2*, *Pfkfb3*, *Eno1*, and *ldhb*, are also upregulated in the partially ligated left carotid artery (LCA, disturbed flow region), compared to the sham-operated right carotid artery (RCA, steady flow region) (Fig. 1*B*). Analyses of published transcriptomic data indicate that OS upregulates glycolytic genes, at least in part, at the mRNA level. To interrogate the role of METTL3 in the OS regulation of glycolytic genes, we knocked down METTL3 and then subjected these ECs to OS. As revealed by the extracellular acidification rate (ECAR), METTL3 knockdown markedly attenuated the OS-elevated glycolysis to a level similar to that in PS-imposed ECs (Fig. 1*C*). Consistently, the OS-elevated lactate level was significantly attenuated in ECs with METTL3 knockdown (Fig. 1*D*). HK1 and PFKFB3 catalyze the rate-limiting steps of glycolysis, whereas GCKR negatively regulates HK1 to inhibit glycolysis. We have previously demonstrated that OS upregulates HK1 and PFKFB3 and downregulates GCKR (4). Thus, we explored whether HK1 and PFKFB3 are positively regulated and GCKR is negatively regulated by the METTL3. As shown in Fig. 1 *E* and *F*, OS increased the mRNA and protein levels of HK1 and PFKFB3, which were largely abolished by METTL3 knockdown. In contrast, OS-suppressed GCKR was reversed by METTL3 knockdown (Fig. 1 *E* and *F*). Together, the results in Fig. 1 suggest that the OS-augmented EC glycolysis was through METTL3 regulation of HK1, PFKFB3, and GCKR.

### Shear Stress Regulation of Glycolytic Genes via m^6^A Modifications.

**Fig. 2.**
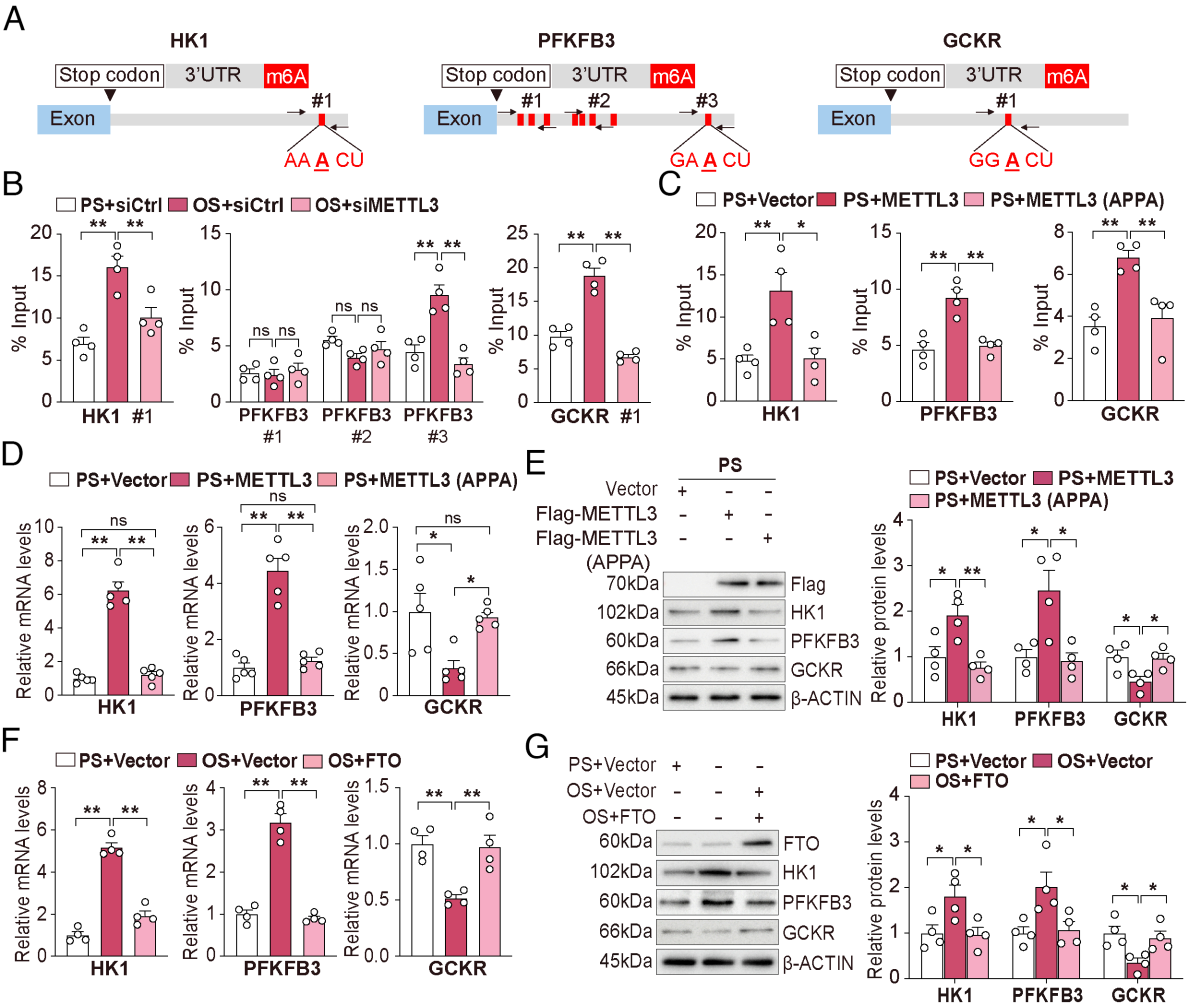
Shear stress regulates glycolytic genes via m^6^A modifications. (*A*) Prediction of the m^6^A methylation sites in HK1, PRKFB3, and GCKR mRNA. (*B* and *C*) m^6^A-RIP-qPCR analysis of m^6^A modified HK1, PFKFB3, and GCKR mRNA in ECs under the indicated conditions (n = 4/group). (*D*) qPCR analysis of the indicated mRNA levels in ECs overexpressing METTL3 or METTL3(APPA) under PS (n = 5/group). (*E*) Western blot and quantification of METTL3, HK1, PFKFB3, and GCKR in ECs overexpressing METTL3 or METTL3(APPA) under PS (n = 4/group). (*F*) qPCR analysis of HK1, PFKFB3, and GCKR in ECs overexpressing FTO or control vector under PS or OS (n = 4/group). (*G*) Western blot and quantification of METTL3, HK1, PFKFB3, and GCKR levels in the indicated groups (n = 4/group). Data are presented as mean ± SEM. *P < 0.05, **P < 0.01.

We further investigated the m^6^A modification in the OS-induced glycolytic genes. Mining our published eCLIP-seq data (2), we confirmed that OS induced the m^6^A modifications of HK1, PFKFB3, and GCKR (2). Our previous study demonstrated that the OS-activated METTL3 predominantly hypermethylates m^6^A sites near the 3’UTR of its target genes (2). We thus used the SRAMP algorithms (21) to predict the high-confidence m^6^A sites in the 3’UTR of HK1, PFKFB3, and GCKR mRNAs (Fig. 2*A*). To validate this in silico prediction, we performed m^6^A-RNA–immunoprecipitation (m^6^A-RNA–IP) qPCR with primers flanking these m^6^A sites. As anticipated, OS enriched m^6^A modification in the 3’UTRs of HK1, PFKFB3, and GCKR mRNAs, which was attenuated by METTL3 knockdown (Fig. 2*B*). In a complementary experiment using ECs under PS, overexpression of wild-type (WT) METTL3, but not the catalytic mutant METTL3-APPA (22), increased the m^6^A modification of these mRNAs (Fig. 2*C*), attendant with increased mRNA and protein levels of HK1 and PFKFB3, but decreased levels of GCKR (Fig. 2 *D* and *E*). Moreover, overexpression of the m^6^A eraser FTO (which reverses the action of METTL3) significantly inhibited the OS-upregulated HK1 and PFKFB3 and reverted the OS-downregulated GCKR (Fig. 2 *F* and *G*). Collectively, the results in Fig. 2 suggest that shear stress regulation of HK, PFKFB3, and GCKR is mediated through METTL3-mediated m^6^A modifications.

### SGLT2i Reduces EC Glycolysis Through Suppression of METTL3.

**Fig. 3.**
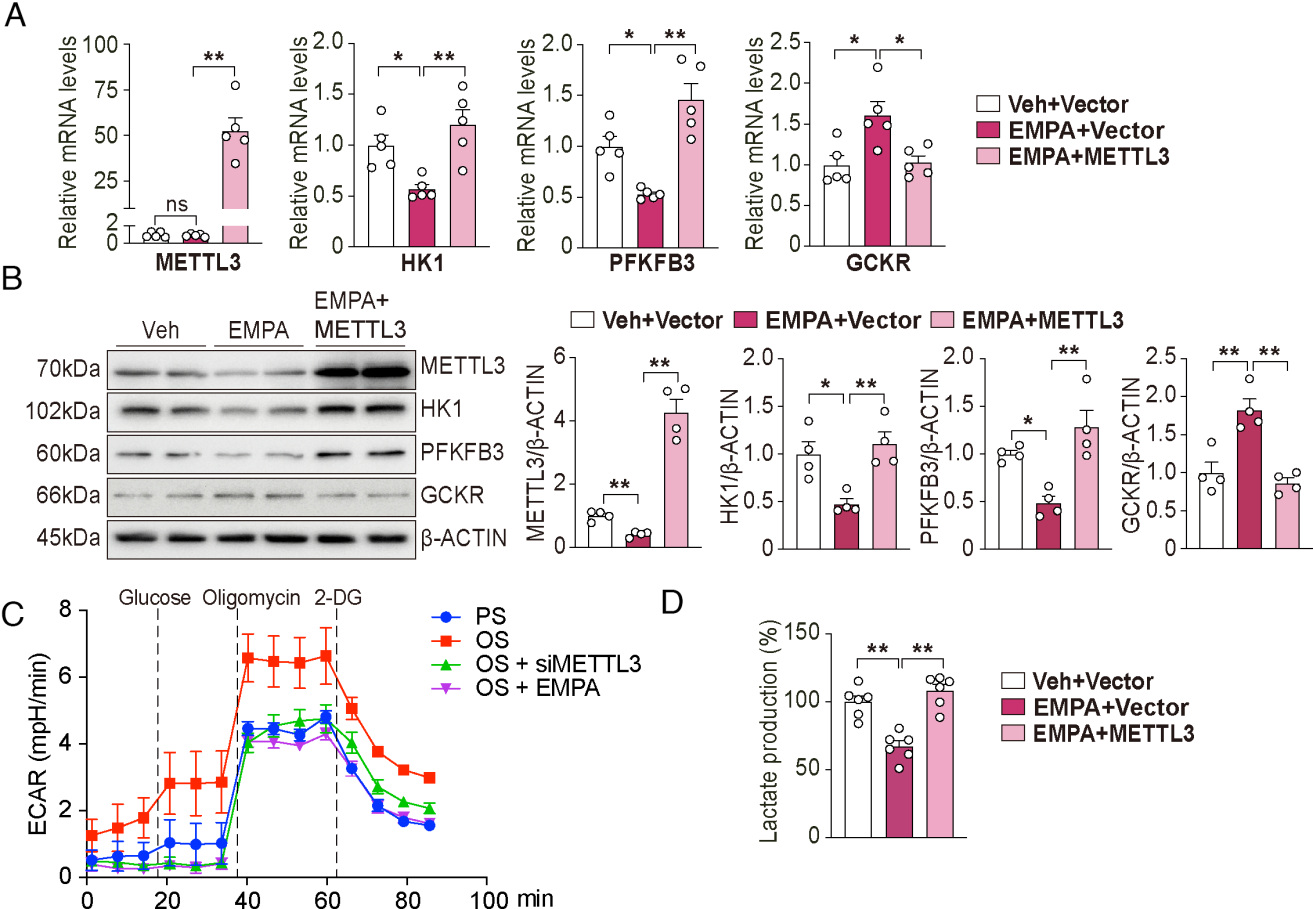
Empagliflozin (EMPA) reduces EC glycolysis via METTL3. (*A*) qPCR analysis of HK1, PFKFB3, and GCKR mRNA expressions in EMPA treated-ECs with/without METTL3 overexpression for 24 h (n = 5/group). (*B*) Western blot and quantification of METTL3, HK1, PFKFB3, and GCKR expressions in EMPA-treated ECs with/without METTL3 overexpression (n = 4/group). (*C*) Seahorse assay measuring ECAR changes in the indicated groups (n = 4/group). (*D*) Measurement of relative lactate content in ECs in the indicated groups (n = 6/group). Data are presented as mean ± SEM. *P < 0.05, **P < 0.01.

With SGLT2 acting as a positive regulator for glycolysis, we further examined whether SGLT2i can act as PS to mitigate EC glycolysis via suppressing METTL3. To this end, we treated ECs with Empagliflozin (EMPA, an SGLT2i), which has been shown to improve cardiovascular outcomes in patients with type 2 diabetes (23). As shown in Fig. 3 *A* and *B*, EMPA treatment significantly reduced the protein level of METTL3, while having little effect on its mRNA level. In line with the decreased level of METTL3, the expression of HK1 and PFKFB3 was reduced and that of GCKR was increased. Furthermore, the effect of EMPA on these glycolytic genes was reversed when METTL3 was overexpressed in ECs (Fig. 3 *A* and *B*). Functionally, ECAR and lactate production assays demonstrated that EMPA decreased EC glycolysis, and this was reverted by the METTL3 overexpression (Fig. 3 *C* and *D*). These data demonstrated that SGLT2i, exemplified by EMPA, attenuated EC glycolysis via downregulation of METTL3.

### OS Induces Glucose Uptake via METTL3.

**Fig. 4.**
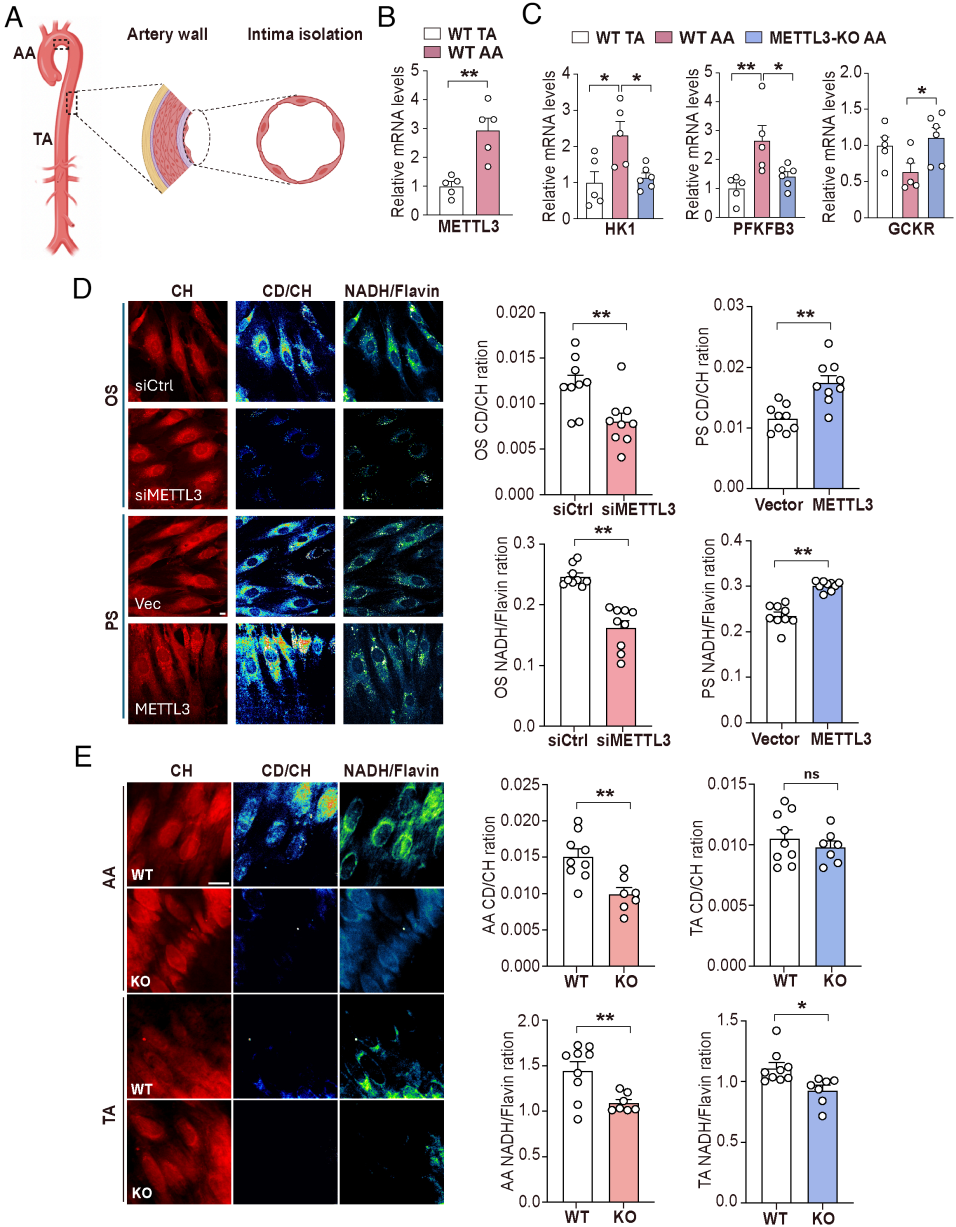
Atheroprone flow increases glycolysis via METTL3 in vivo. (*A*) Schematic diagram of vascular intima isolated from the mouse TA and AA; (*B*) qPCR analysis of Mettl3 mRNA expression in the intima from TA and AA of C57BL/6 J WT mice; (*C*) qPCR analysis of Hk1, Pfkfb3, and Gckr mRNA expressions in the intima of the TA or AA from WT (Mettl3-floxed) and Mettl3 KO mice; (*D*) ECs were treated with 25 mM D7-glu for 72 h and then subjected to OS for 48 h, followed by SRS imaging analysis for CD/CH and NADH/Flavin for the assessment of lipid turnover and redox ratio, respectively. (Scale bar, 10 µm.) n = 9; data are presented as mean ± SEM. *P <0.05; **P <0.01 between indicated groups. (*E*) Mettl3 KO and WT (Mettl3-floxed) mice were fed D7-glu water for 2 wk. The TA and AA regions of the arterial tree were subjected to SRS imaging analysis ex vivo for CD/CH lipid turnover rate and NADH/Flavin redox ratio. (Scale bar, 10 µm.) n = 7 for METTL3 KO mice and n = 9 for WT mice; data are presented as mean ± SEM. *P <0.05, **P <0.01 between indicated groups.

After establishing the OS/PS regulation of glycolysis via m^6^A modification by METTL3, we investigated the roles of flow-regulated METTL3 and the epitranscription of glycolytic genes in EC metabolism in vivo. Isolated aortic arch (AA) and thoracic artery (TA) intima were used to represent the vascular segments under atheroprone and atheroprotective flow, respectively (Fig. 4*A*). qPCR analysis confirmed that METTL3 was increased in the AA intima compared to the TA intima (Fig. 4*B*). Additionally, mRNA levels of Hk1 and Pfkfb3 were higher, while that of Gckr was lower, in the AA intima compared to those in the TA intima (Fig. 4*C*). Importantly, the expression levels of Hk1, Pfkfb3, and Gckr in the AA and TA intima were reverted in EC-METTL3^−/−^ mice in comparison with those in WT mice (Fig. 4*C*). These results demonstrate that METTL3 mediates atheroprone flow–induced glycolysis in vivo.

### SRS Imaging Analysis of Glucose Metabolism In Vitro and In Vivo.

Next, we performed experiments using D7-glu labeled ECs to assess the role of METTL3 in regulating EC metabolism under flows. ECs were transfected with METTL3 or siMETTL3 and subjected to PS or OS. The CD signals were significantly higher in ECs under OS, and the elevation was abolished by siMETTL3. On the other hand, ECs overexpressing METTL3 showed increased CD signals under PS. These results indicated that Mettl3 increased de novo synthesized lipids that were derived from the intaked D7-glu (Fig. 4*D*).

To further investigate the glucose metabolites in the vascular wall in mice with genetically manipulated Mettl3, we fed EC-Mettl3^−/−^ and their WT littermate mice with water containing 3% D7-glu for 2 wk and then monitored the glucose metabolism in vivo. SRS imaging demonstrated a higher CD/CH ratio in the atheroprone AA region, indicating elevated D7-glu incorporation into de novo synthesized lipids in AA, compared to the atheroprotective TA region (Fig. 4*E*). The NADH/flavin ratio was also significantly higher in AA than TA (Fig. 4*E*), suggesting increased catabolism in the atheroprone areas. However, these metabolic elevations were compromised in Mettl3 knockout mice. Taken together, we used SRS imaging to show that the atheroprone flow elevated glucose uptake and metabolism in vitro and in vivo via the METTL3-mediated mechanism.

## Discussion

Flow disturbance significantly impacts EC functions by increasing glycolysis (24–26). Recent studies have highlighted m^6^A methylation of RNA as a crucial epitranscriptomic mechanism in ECs (2, 27). METTL3, a key member of the m^6^A “writer” methyltransferase complexes, is upregulated under OS (2). Additionally, METTL3-dependent m^6^A modifications have been reported to regulate glycolysis in various pathophysiological processes (27). In this study, we present evidence that OS increases METTL3 expression to enhance m^6^A modifications of mRNAs of several key glycolysis genes HK1, PFKFB3, and GCKR. Such augmented m^6^A modifications increase the expressions of HK1 and PFKFB3 while inhibiting GCKR expression, which results in elevated EC glycolysis. Moreover, we demonstrate that EMPA, a widely used SGLT2i, represses METTL3 expression, thereby attenuating the OS-induced glycolysis in ECs.

Compared to other cell types that utilize the Krebs cycle to generate ATP, ECs rely highly on glycolysis to produce cellular ATP (28). Homeostatic ECs are generally quiescent, with moderate demands on glycolysis. However, during the onset of diseases such as atherosclerosis, pulmonary arterial hypertension (PAH), cancer, and diabetes, ECs shift toward the Warburg effect, characterized by the augmented glycolytic flux (29). Altered glycolysis induces EC dysfunction by disrupting energy metabolism, producing metabolic byproducts like lactate and protons that lead to cellular acidification, and generating reactive oxygen species that cause oxidative stress (30). Additionally, dysregulated glycolysis impairs nitric oxide production, reduces its bioavailability, and can trigger inflammatory pathways (31). Enhanced glycolysis is also associated with the endothelial-to-mesenchymal transition, contributing to vascular stiffness and fibrosis (3). HK1 and PFKFB3 are important rate-limiting enzymes in the early steps of glycolysis (32, 33). Studies have demonstrated their critical roles in EC dysfunction. Inflammatory and oxidative stresses increase EC glycolysis by upregulating the expression and activity of HK1 and PFKFB3 (32, 33); consistent with our results, different flow conditions can differentially regulate HK1 and PFKFB3. While PS decreases EC glycolysis through KLF2-mediated suppression of PFKFB3 (5), OS and hypoxia increase EC glycolysis, partly through HIF-1α-mediated induction of HK1 and PFKFB3 (32, 34–36). Additionally, glycolysis is inhibited by the binding of GCKR to HK1, which sequesters HK1 in the nucleus (37). We have previously demonstrated that PS markedly upregulates GCKR, leading to the suppression of HK1 and decreased glycolysis (4). Herein, we show that OS significantly downregulates GCKR, in part through METTL3. Thus, METTL3 plays a crucial role in linking EC mechanotransduction with metabolic reprogramming via epitranscriptional regulation.

Our previously published data indicate that OS prominently increases METTL3-dependent m^6^A methylation in ECs (2). This study demonstrates that METTL3 markedly enhances glycolysis by promoting m^6^A modification of HK1, PFKFB3, and GCKR, suggesting that METTL3 exacerbates the OS-induced EC dysfunction. Consistent with our results, a causative role of METTL3 in EC dysfunction has been previously identified (38). For example, METTL3 can stabilize or increase the translation of mRNAs encoding glycolytic enzymes HK2, pyruvate dehydrogenase kinase 4, and enolase 1 (39), thereby enhancing cancer cell glycolysis and tumorigenesis (40, 41). As well, OS induces METTL3, which increases NLRP1 and decreases KLF4 mRNAs, leading to EC inflammatory responses, including NF-κB activation and monocyte adhesion (2). METTL3 knockdown attenuates neointima formation in the partially ligated carotid artery in mice, indicating that OS induction of METTL3 is atheroprone (2). Noticeably, we observed that METTL3 upregulates HK1 and PFKFB3 while downregulating GCKR. RNA m^6^A editing is tightly regulated by RNA writers (e.g., METTL3, METTL14, WTAP1, and KIAA1429), readers (e.g., YTHDF1-3, YTHDC1, and YTHDC2), and erasers (e.g., FTO and ALKBH5), with the “readers” determining the regulation of gene expression by m^6^A modification (8). Therefore, METTL3 plays a distinct role in modulating the expression of HK1 and PFKFB3 vs. GCKR under flow. These opposite regulation mechanisms may be attributed to the recruitment of distinct readers to target mRNAs.

Our study also reveals that SGLT2i inhibits METTL3 to decrease EC glycolysis. SGLT2i lowers blood glucose levels by inhibiting SGLT2 in the kidney, which indirectly affects glycolysis by altering glucose availability and energy metabolism (42). By promoting glucose excretion, SGLT2i reduces the amount of glucose available for glycolysis in the liver and skeletal muscles (43, 44). This reduction in glucose availability can lead to increased reliance on alternative energy sources, such as fatty acids and ketone bodies, thereby shifting the metabolic balance away from glycolysis (43, 44).

We provide evidence that EMPA, a commonly prescribed SGLT2i, represses glycolysis by downregulating METTL3 and potentially suppressing METTL3-mediated m^6^A modification of HK1, PFKFB3, and GCKR. Thus, SGLT2i and atheroprotective flow synergistically downregulate METTL3. Whether other atheroprotective factors benefit EC function via quenching glycolysis warrants further investigation. The mechanisms by which EMPA regulates METTL3 protein remain unclear. However, SGLT2 inhibitors can regulate the posttranslational modifications, including phosphorylation, glycation, and ubiquitination (45–47). Therefore, EMPA might affect METTL3 via posttranlational modifications such as ubiquitination.

**Fig. 5.**
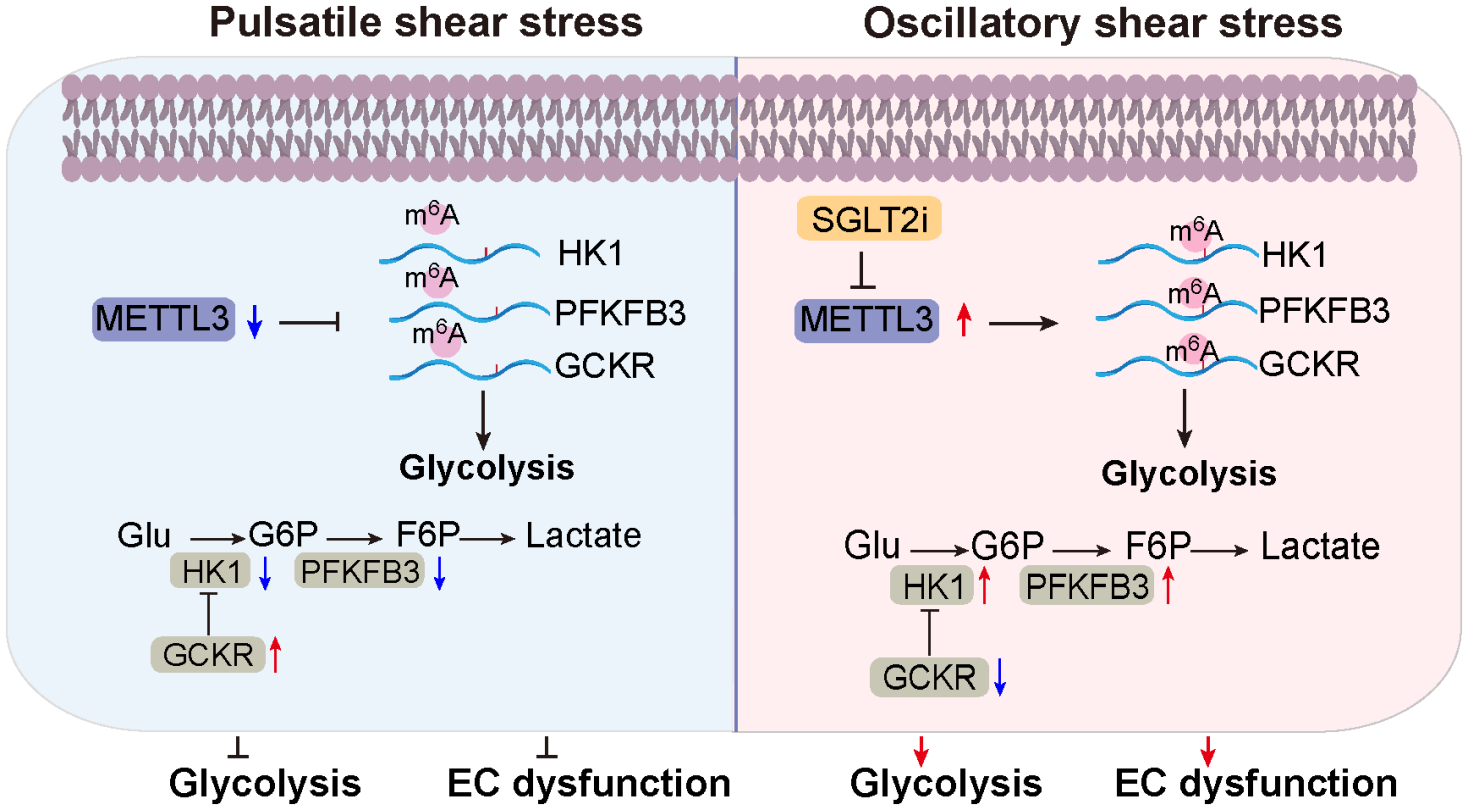
Schematic illustration showing that atheroprone flow increases glycolysis in ECs via METTL3. Atheroprotective flow such as PS suppresses the expression of METTL3, resulting in low levels of m^6^A modification of the glycolysis genes HK1, PFKFB3, and GCKR. Such regulation of glycolysis maintains EC homeostasis. Under atheroprone flow such as OS, METTL3 expression is increased, leading to enhanced m^6^A modifications of HK1, PFKFB3, and GCKR. These augmented m6A modifications upregulate HK1 and PFKFB3 expressions while inhibiting GCKR expression, contributing to elevated glycolysis and EC dysfunction.

Summarized in Fig. 5, this study uncovers mechanisms by which atheroprone flow promotes EC glycolysis through the induction of METTL3. On the other hand, atheroprotective flow and SGLT2i repress glycolysis by inhibiting METTL3-m^6^A modifications of HK1, PFKFB3, and GCKR. These findings enhance our understanding of EC biology in health and disease by connecting epitranscriptional state to metabolic regulation.

## Materials and Methods

### Animals.

Animal experiments were approved by the Institutional Animal Care and Use Committee of the University of California, San Diego. All rodent models were maintained on a 12 h light/dark cycle and fed ad libitum with a chow diet. To generate endothelial-specific METTL3 knockout mice, homozygous floxed METTL3 mice (METTL3^flox/flox^) were crossed with VE-Cadherin Cre mice on a C57BL/6 background. Eight- to 10-wk-old male EC-METTL3^−/−^ mice and their WT littermates were used for experiments.

### Cell Culture and Transfection.

Human umbilical vein endothelial cells (HUVECs) purchased from Cell Applications were cultured in M199 medium (Gibco) supplemented with 15% fetal bovine serum (FBS) (Gibco), 10% Clonetics endothelial growth medium, 1% L-glutamine, 1% sodium pyruvate, and 100 U/mL penicillin-streptomycin. ECs at passages four to six were used for the subsequent experiments.

Plasmids were transfected by electroporation. Briefly, HUVECs were harvested and resuspended in an electroporation buffer (Lonza). Plasmids were then added to the cells and mixed well. The A-034 program and electroporation device (Lonza, Amaxa 4D-Nucleofector) were used for transfection, and the cells were then seeded on collagen I-coated slides or culture plates. For siRNA transfection, HUVECs were transfected with METTL3 siRNA (Thermo Fisher Scientific) or scramble control RNA at 50 nmol/L using the Lipofectamine RNAiMAX Transfection Reagent (Invitrogen).

### Flow Experiments.

Flow experiments were performed using a circulating flow system containing a parallel-plate flow chamber to apply pulsatile shear stress (PS, 12 ± 4 dyn/cm^2^) or oscillatory shear stress (OS, 0.5 ± 4 dyn/cm^2^) to the HUVECs. The flow system was maintained at 37 °C and ventilated with 95% humidified air and 5% CO_2_.

### qPCR.

Total RNA was isolated from cultured ECs or the intima of the mouse AA and TA using the RIzol reagent (Invitrogen). The extracted RNA was reverse-transcribed into cDNA using the PrimeScript RT (TaKaRa,). Real-time PCR was conducted with SYBR Green real-time PCR master mix (Thermo Fisher Scientific) on a CFX Connect™ real-time PCR detection system (BioRad). The 2^-∆∆Ct^ method was used to calculate results, and mRNA expression levels were normalized to β-actin expression. All primers used are listed in *SI Appendix*, Table S1.

### Western Blot.

For western blot analysis, ECs or vascular intima tissues were lysed in RIPA lysis buffer supplemented with protease inhibitors (Thermo Fisher Scientific). Protein concentrations were quantified using the BCA Protein Assay Kit (Thermo Fisher Scientific). After adjustment to the same concentration, equal amounts of protein were separated by SDS-PAGE and transferred to PVDF membranes (Cytiva). The membranes were then incubated with the indicated primary antibodies at 4 °C overnight, followed by incubation with HRP-conjugated secondary antibodies at room temperature for 1 h. Protein bands were visualized using the ECL system (Bio-Rad). The following primary antibodies were used: METTL3 (Cell Signaling Technology), HK1 (Cell Signaling Technology), PFKFB3 (Cell Signaling Technology), GCKR (Cell Signaling Technology), and β-actin (Cell Signaling Technology).

### ECAR Assay.

ECs were seeded into Seahorse XF96 cell culture microplates (Agilent) at a density of 1.0 × 10^4^ cells per well and incubated overnight or 4 h for static and flow experiments, respectively. The medium was then replaced with Seahorse XF Base Medium (Agilent) supplemented with 1 mM pyruvate and 2 mM L-glutamine, and the cells were equilibrated in a non-CO2 incubator for 1 h. ECAR was measured using the Seahorse XF96 Extracellular Flux Analyzer (Agilent). Baseline ECAR was recorded, followed by the sequential addition of glucose (Gibco, 10 mM) to stimulate glycolysis, oligomycin A (MedChemExpress, 1 µM) to inhibit ATP synthase and reveal the maximum glycolytic capacity, and 2-Deoxy-D-Glucose (Sigma-Aldrich, 50 mM) to halt all glycolytic acidification.

### Lactate Assay.

Lactate concentrations in cell lysates were determined using the L-Lactate Assay Kit (Cayman Chemical) according to the manufacturer’s instructions. Briefly, 50 μL of each sample or lactate standard was added to individual wells of a 96-well microplate. To each well, 100 μL of Assay Buffer was added, followed by 50 μL of Cofactor Preparation, 10 μL of Enzyme Mix, and 50 μL of Dye Reagent. The reaction mixture was gently mixed and incubated at room temperature for 30 min in the dark. The absorbance was measured at 540 nm using a microplate reader. The lactate concentration in the samples was calculated by plotting the absorbance values against the known concentrations of lactate standards.

### m^6^A-IP and qPCR.

m^6^A-IP was conducted as previously described (48). Briefly, RNA samples were fragmented using a fragmentation reagent (Invitrogen, AM8740) and then incubated with Dynabeads Protein G (Invitrogen) and an m^6^A antibody at 4 °C overnight. After incubation, the beads were washed twice with 900 μL of cold high-salt wash buffer (50 mM Tris-HCl pH 7.4, 1 M NaCl, 1 mM EDTA, 1% NP-40, 0.1% SDS, 0.5% sodium deoxycholate, 1,000 U/mL RNase inhibitor), then washed twice with 900 μL of cold wash buffer (20 mM Tris-HCl pH 7.4, 10 mM MgClâ‚‚, 0.2% Tween-20, 1,000 U/mL RNase inhibitor). After washing, the methylated RNAs were eluted with an elution buffer (6 mM m^6^A sodium salt, 50 mM Tris-HCl, 150 mM NaCl, 1 mM EDTA, 1% NP-40, 1 U/μL RNase inhibitor). The RNAs were then isolated using the RNeasy Kit (Qiagen), and the m^6^A-methylated RNA was quantified using the One-Step Real-Time RT-PCR Kit (Invitrogen).

### SRS Imaging.

For in vitro SRS imaging, HUVECs were cultured with M-199 media containing 25 mM D7-glu for 72 h for metabolic labeling and then subjected to OS or PS for 48 h in M-199 media containing 25 mM D7-glu. For the in vivo SRS imaging assay, C57BL/6 mice were fed 3% D7-glu water for 2 wk. After labeling, the mice were anesthetized with isoflurane, and the TA and AA regions were surgically exposed. The SRS images of the cells and tissues were obtained using an upright laser-scanning microscope (DIY multiphoton, Olympus) equipped with a synchronized pulsed pump beam (tunable 720 to 990 nm wavelength, 5 to 6 ps pulse width, and 80 MHz repetition rate) and a Stokes beam (wavelength at 1,032 nm, 6 ps pulse width, and 80 MHz repetition rate). The signal peaks at 2,120 cm^−1^ for the uptake of D7-glu, and at 2,125, 2,150, 2,172, and 2,200 cm^−1^ for the de novo synthesis of lipids and proteins from D7-glu-derived metabolites were analyzed. Analyses of glucose metabolic changes were determined by signals of NADH/Flavin redox ratio at corresponding Raman shifts, and the lipid turnover rates were determined by the ratios of CH (the vibrational signal of the carbon-hydrogen bond) and CD (the vibrational signal of the carbon-deuterium bond) signals. SRS imaging at 2,150 cm^−1^ of cells labeled with [D7]-glucose indicates time-dependent SRS intensity of newly synthesized C–D bonds in the biomass. Imaging at 2,930 cm^−1^ of CH3 vibrational intensity represents the total protein and lipid signals. CD/CH indicates the percentage of newly synthesized biomass to total biomass, thus the turnover of biomass in cells (49). Images were processed and quantified using ImageJ software.

### Statistical Analyses.

All data are expressed as mean ± SEM. A 2-tailed Student’s *t* test was used to compare the means of two groups with normal distribution, while a nonparametric test (Mann–Whitney U test) was applied for data with skewed distribution. One-way ANOVA with a Bonferroni post hoc test (assuming equal variances) or Tamhane’s T2 test (not assuming equal variances) was used to compare the means of multiple groups. All statistical analyses were performed using Statistical Package for the Social Sciences software. Statistical significance was considered if the *P* value was less than 0.05.

## Supplementary Material

Appendix 01 (PDF)

## Data Availability

All study data are included in the manuscript and/or *SI Appendix*.
