# Supplementary material for: METTL3 mediates atheroprone flow–induced glycolysis in endothelial cells
Source: Proc Natl Acad Sci U S A. 2025 May 6;122(19):e2424796122. doi: 10.1073/pnas.2424796122 (PMC12088407; doi:10.1073/pnas.2424796122)
Supplement: Supplementary file 1 — Appendix 01 (PDF) [file pnas.2424796122.sapp.pdf]

PNAS

Supporting Information for
METTL3 Mediates Atheroprone Flow-induced Glycolysis in Endothelial Cells

Guo-Jun Zhao[a,b], So Yun Han[b], Yajuan Li[c,d], Dongqiang Yuan[e], Shuo Qin[c], Yuhan Li[c], Hongje Jang[c], Li-Jing Chen[d], Tong-You Wade Wei[b], Ming He[b], Yi-Shun Li[c,d], Zhen Bouman Chen[e], Lingyan Shi[c], Shu Chien[c,d,*], and John Y-J Shyy[b,d,*]

[a] Department of Cardiology, The First Affiliated Hospital of Zhengzhou University, Zhengzhou, 450052, China; [b] Division of Cardiology, Department of Medicine, University of California, San Diego, La Jolla, CA, 92093; [c] Department of Bioengineering, University of California at San Diego, La Jolla, CA 92093; [d] Institute of Engineering in Medicine, University of California, San Diego, La Jolla, CA 92093; [e] Department of Diabetes Complications and Metabolism, Beckman Research Institute, City of Hope, CA 91010

***Corresponding authors**:

Shu Chien, MD. Ph.D., Email: shuchien@ucsd.edu

John Y-J. Shyy, Ph.D., Email: jshyy@health.ucsd.edu

This PDF file includes:

Table S1

Table S1. Primers used for qPCR.

Gene name	Forward	Reverse
Human METTL3	TTGTCTCCAACCTTCCGTAGT	CCAGATCAGAGAGGTGGTGTAG
Human HK1	CCAACATTCGTAAGGTCCATTCC	CCTCGGACTCCATGTGAACATT
Human PFKFB3	ATTGCGGTTTTCGATGCCAC	GCCACAACTGTAGGGTCGT
Human GCKR	CAAGTGGGAGTTGTCTGGGTA	TGCCCTAGCAGTCGAACAATG
Human β-actin	CATGTACGTTGCTATCCAGGC	CTCCTTAATGTCACGCACGAT
Mouse METTL3	CTGGGCACTTGGATTTAAGGAA	TGAGAGGTGGTGTAGCAACTT
Mouse HK1	CGGAATGGGGAGCCTTTGG	GCCTTCCTTATCCGTTTCAATGG
Mouse PFKFB3	CCCAGAGCCGGGTACAGAA	CAACTCCCCAACCGTGATTGT
Mouse GCKR	CCAAGCACCAAGCGGTATCA	GTCAGTGGGTTGGACTTCTCT
Mouse β-actin	GGCTGTATTCCCCTCCATCG	CCAGTTGGTAACAATGCCATGT
**Primers used for m6A-IP and qPCR**		
	**Forward**	**Reverse**
HK1 #1	CAGTCCTGCGGAAATGTGTC	TAGGTCATACGACACGGCT
PFKFB3 #1	CCCGAGGCAAAACGTATCCT	GGTGCCAAGCATGGTTCTCT
PFKFB3 #2	AACCTCCACGTGACTGTTTG	ACACCTGTCCAAGTTCGTTC
PFKFB3 #3	CACGGGACTTTTAGTTTGCG	TCACTGGCTTGCAAAGTAGG
GCKR #1	CATGTTTCTGGGTGGGTGAA	TCCCCCAAAGTGGAGAGAAT
